# Innovations, Challenges, and Future Prospects for Combination Vaccines Against Human Infections

**DOI:** 10.3390/vaccines13040335

**Published:** 2025-03-21

**Authors:** Munazza Fatima, Kee-Jong Hong

**Affiliations:** 1 Department of Microbiology, Gachon University College of Medicine, Incheon 21936, Republic of Korea; munazzafatima@gachon.ac.kr; 2 Lee Gil Ya Cancer and Diabetes Institute, Gachon University, Incheon 21999, Republic of Korea; 3 Department of Health Sciences and Technology, GAIHST, Gachon University, Incheon 21999, Republic of Korea; 4 Korea mRNA Vaccine Initiative, Gachon University, Seongnam 13120, Republic of Korea

**Keywords:** combination vaccines, multivalent vaccine, influenza, SARS-CoV-2, mRNA, recombinant, viral vector, attenuated

## Abstract

Combination vaccines provide the versatile benefits of addressing different pathogens simultaneously using a combined formulation. This approach can be regarded as a substantial modernization in immunization. In this review, we highlight various advancements in combination vaccines based on mRNA, viral vectors, live attenuated, and recombinant vaccines. Recent success in clinical trials of mRNA platforms for combination vaccines has particularly accelerated research in this direction. The advantages of combination vaccines in terms of patient adherence, cost effectiveness, and streamlined immunization schedule are discussed. The existing challenges of antigenic interference, logistical hurdles, and the complications of regulatory standards are analyzed. Research trends to make combination vaccines viable for emerging infections have been summarized. The current work provides a critical overview, the existing opportunities, and the future prospects of combination vaccines.

## 1. Introduction

Vaccines are vital in raising immunity against diverse infections. They provide one of the most reliable tools and affordable solutions to maintain public health. According to an estimate, vaccination has saved almost 154 million lives in the last five decades, equivalent to about six lives every minute [1]. Global immunization has resulted in a nearly 40% reduction in infant mortality by protecting against infectious diseases. These include bacterial infections such as diphtheria, pertussis, tetanus, Meningitis A, Haemophilus influenzae Type B, invasive pneumococcal and tuberculosis, and viral infections such as polio, measles, rubella, rotavirus, Hepatitis B, Japanese encephalitis, and yellow fever [2]. Vaccination is also helpful in overcoming the issue of antimicrobial resistance by reducing the usage of antibiotics [3]. According to Humphries and Bystrianyk in the book Dissolving Illusion, improvements in living standards, including housing, clean water, proper nutrition, and sanitation have contributed significantly to the reduction in infectious diseases, rather than vaccines. They claimed that polio mortality declined before vaccines because of improved living standards [4]. Historical evidence counters the claim that socioeconomic factors are the sole drivers of disease decline. For instance, Harrison highlighted historical data showing trends of increasing polio incidence before vaccination and a rapid reduction after vaccine introduction. According to Harrison, better medical care may have decreased polio mortality, but polio cases and paralysis increased, and only vaccination effectively controlled polio [5]. Furthermore, the near eradication of polio has been achieved despite poverty in endemic regions. In 1998, the Global Polio Eradication Initiative (GPEI) was launched, resulting in a 99% reduction in polio cases, which has spared an estimated 20 million children. This indicates the vital role of vaccines in reducing disease transmission and mortality [6,7,8]. The irreplaceable role of the polio vaccine was highlighted during the recent COVID-19 pandemic, where disruptions in polio immunization led to the reemergence of polio in areas that were previously declared polio-free [9]. Another success story of the transformative role of vaccines is the eradication of smallpox. Despite hygiene and medical care improvements, smallpox remained a threat globally. In 1967, WHO launched the Intensified Smallpox Eradication Program (ISEP), combining mass vaccination (80%) coverage and surveillance for outbreak containment. By 1977, the last case was reported in Somalia, and 1980, the WHO declared smallpox eradication [10,11]. This success countered the diffusion illusion and reinforced vaccines’ significance.

**Figure 1:**
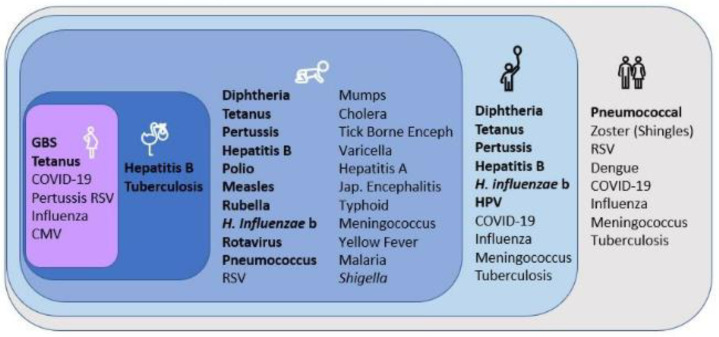
Pathogens and diseases targeted by vaccines across different age groups. Targets of WHO-recommended vaccines for routine immunization are shown in bold. Others are recommended for specific contexts, regions, populations, or vaccines in clinical trials (adapted from [15]).

Combination vaccines address multiple infections by providing a broader immune response with a single dose [12]. Administration of multiple antigens simultaneously provides a simplified strategy for enhancing vaccination rates. It may ease vaccination schedules and ensure patient compliance. The development of combination vaccines is mainly driven by a high disease burden originating from multiple infections. This strategy may lead to a significant drop in disease burden [12]. It is particularly beneficial for resource-limited countries, as it reduces logistical challenges [13]. In the past decade, WHO recommended vaccines targeting high-burden regions and high-risk populations, such as dengue and typhoid. Along with these, some global recommendations for RSV and tuberculosis may further increase the required immunizations for children, especially in LMICs. Concerns over multiple injections and visits may result in delaying or missing doses, leaving children prone to infections. By 2030, a vaccine is expected to be recommended for up to 30 infections, as shown in Figure 1. Combination strategies will be crucial in optimizing immunization programs as new vaccines emerge, with minimal logistical burdens. Studies by PATH found that stakeholders and health care providers prefer combination vaccines over adding new standalone vaccines. Recent developments in mRNA technology have revealed new possibilities for combination vaccine production, allowing rapid adjustments to target many infections [14]. Although there have been impressive advancements, existing challenges hinder the widespread application of combination vaccines. These challenges include maintaining efficacy, addressing safety concerns, and ensuring optimal compatibility among vaccine components. It requires meticulous preclinical studies and clinical validation to ensure the noninferiority and safety of a combination. Despite hurdles, the quest for developing combination vaccines through ongoing research and development is crucial for improving global health.

## 2. Currently Used Combination Vaccines

The immune system is efficiently responsive to several antigens without being overburdened. For instance, the DTaP vaccine can protect against diphtheria, tetanus, and pertussis simultaneously. This combination vaccine plays a vital role in public health efforts, significantly reducing disease occurrence and the associated life-threatening complications [16]. Before the introduction of the diphtheria vaccine, cases had already declined by 80% due to improvements in hygiene, sanitation, and medical care [4]. However, despite this reduction, the disease caused 21,053 cases and 1822 deaths annually from 1936 to 1945. After the introduction of vaccination, the number of cases dropped drastically. By 2006, diphtheria was eradicated in the U.S., with zero cases and deaths reported [17]. Vaccination is essential for interrupting disease transmission and preventing its re-emergence [18,19]. Recently reported cases show that diphtheria remains endemic in regions where immunization rates are low [20,21]. In Africa, over 65% of diphtheria cases are reported in unvaccinated individuals. The outbreak is driven by low herd immunity caused by suboptimal vaccine uptake [22]. A study in Senegal, West Africa, reported high morbidity and mortality from pertussis before vaccination, with an incidence of 183 cases per 1000 children and a mortality rate of 2.8%. After introducing the vaccine, the incidence of pertussis declined by 27% over three years and by 46% over six years, underscoring the benefit of vaccination [23]. The transition to acellular vaccines improved safety, but recent resurgences in countries using DTaP have raised concerns about its long-term effectiveness [24,25]. Some studies raised safety concerns about DTaP vaccines, specifically in low-income countries [26,27]. However, WHO has concluded that there is sufficient evidence to dismiss the hypothesis that vaccination increases non-specific mortality [28]. Combining antigens does not enhance unfavorable effects but can actually minimize them [29]. The MMR vaccine has proven safety and effectiveness against measles, mumps, and rubella [30]. Studies indicate that initial dose of the MMR vaccine provides 97% efficacy against rubella, 93% efficacy against measles, and a slightly lower 78% efficacy against mumps. After the secondary dose, the efficacy increases to 97% against measles and 88% against mumps. However, immunity against mumps may decrease over time, and an additional dose could be required, especially in high-risk groups [31]. The addition of the varicella antigen in the MMR vaccine, thus forming the MMRV vaccine, has enhanced its worth, demonstrating high rates of seroconversion against all antigens, with 94.1% efficacy against varicella [32]. The ACIP suggests MMR and varicella or the MMRV vaccine for the first dose at 12–47 months. For the second dose (15 months–12 years) or the first dose at ≥48 months, MMRV is generally preferred over separate injections [33,34]. The MMR and MMRV vaccines are widely regarded as safe and effective but debates about their use and potential adverse effects continue. Concerns about the development of disorders such as autism or bowel disease due to the MMR vaccine have been thoroughly investigated, and expert assessments have continually found no scientific evidence to support these claims. This reinforces the safety and dependability of current vaccination regimens [35].

Research conducted in South Korea on a pentavalent vaccine, DTaP–IPV–Hib, demonstrated that most adverse events were self-limiting, supporting the safety of the vaccine in practical applications [36]. The hexavalent vaccine is currently the most complex vaccine, containing six diverse antigens. It has various formulations that have proven robust immunogenicity without interference, supporting its global usage [37]. Experience of 8 years has revealed that the hexavalent DTaP–HBV–IPV/Hib (Infanrix hexa™) is immunogenic and safe in various vaccination schedules. Its efficacy is comparable with individual vaccines. It can be administered with other vaccines [38]. The immunogenicity of another hexavalent DTwP–HepB–IPV–Hib combination vaccine showed a robust immune response, achieving high seroprotection rates across all included antigens [39]. A recently conducted Phase 3 trial demonstrated that the vaccine’s immunogenicity is comparable with other approved vaccines, with no safety concerns [40]. Approved in 2012, the hexavalent DTaP–IPV–HB–Hib vaccine, known as Hexaxim, has gained worldwide acceptance, with 180 million doses administered globally. It is included in immunization programs across various countries as both a primary vaccine series and a booster dose [41,42]. Currently, several combination vaccines have been authorized globally, with guidelines tailored to address the unique epidemiological challenges of each region. Table 1 outlines various combination vaccines currently used for immunization, along with their target pathogens.

## 3. Upcoming Combination Vaccine Innovations

### 3.1. Combined mRNA Vaccines

The development of mRNA vaccines accelerated during the COVID-19 pandemic, with Moderna and Pfizer-BioNTech setting benchmarks for efficacy and scalability in combating SARS-CoV-2. Researchers are making significant progress in developing mRNA vaccines that target both SARS-CoV-2 and influenza viruses. In particular, Moderna’s Phase 3 trial of mRNA-1083 showed strong immune responses against the influenza virus and SARS-CoV-2 in adults aged 50 years and older. These responses were higher than those generated by licensed flu and COVID-19 vaccines, and the vaccine was found to have acceptable safety and tolerability [45]. In BioNTech’s Phase 3 trial, a robust immune response against Influenza A was observed, and the immune response against Influenza B was lower. However, the immune response against SARS-CoV-2 was comparable with that produced by a licensed COVID-19 vaccine. Additionally, no safety concerns were reported [46]. Studies based on clinical trials have shown promising efficacy of combination vaccine against human metapneumovirus and parainfluenza virus Type 3 [47]. An overview of the promising candidates for next-generation combination vaccines has been given in Table 2. At the same time, extensive preclinical studies are being conducted to optimize the formulation, delivery, and immune responses of mRNA-based combination vaccines. FLUCOV-10, a novel 10-valent mRNA vaccine, has been developed to combat influenza and COVID-19 threats. Hemagglutinin and spike proteins of influenza and SARS-CoV-2 have been used to design vaccines. Preclinical results have shown protection against respiratory infections [48]. In addition, The AR-CoV/IAV mRNA vaccine, which encodes the HA antigen of the H1N1 Influenza A virus and the RBD of the SARS-CoV-2 spike protein, has shown robust immune responses and protection against coinfection with Influenza A and the SARS-CoV-2 variants in animal studies [49]. Another mRNA-based influenza and SARS-CoV-2 combination vaccine has shown no interference between the individual vaccines. The combined vaccine demonstrated adequate protection in mouse studies without evidence of significant interference between individual components. In mice, the vaccine resulted in 100% survival against H1N1 infection and showed efficacy against SARS-CoV-2 [50]. The mRNA-based combination vaccine against bacterial infections such as diphtheria, tetanus, and pertussis is being investigated. Preclinical trials of mRNA-DTaP vaccines have shown promising immunity characterized by robust antibodies and T-cell responses [51]. The WHO/MPP mRNA technology transfer program is developing reliable technology to develop mRNA-based combination vaccines for flaviviruses like dengue and Zika [52]. Although immense efforts are devoted towards mRNA combination vaccines, real-time achievements made so far are still in their infancy. The swift deployment of mRNA COVID-19 vaccines was essential in response to the pandemic, but concerns have been raised regarding safety, efficacy, and regulatory oversight [53]. The mRNA vaccines are associated with waning immunity, lack of sterilizing immunity, and interferon suppression, which may indicate that individuals are not protected from reinfection [54]. The complete N1-methylpseudouridine (m1Ψ) modification may lead to ribosomal frameshifting, translation errors, and cancer risks, highlighting the significance of sequence optimization for safer mRNA therapeutics [55]. The effective delivery of mRNA vaccines relies on lipid nanoparticles, but challenges related to lipid nanoparticle degradation, systemic distribution, and cytotoxicity emphasize the necessity of refining delivery methods to enhance safety and effectiveness [56]. These findings highlight the need for extensive post-market surveillance, risk assessments, and refinement of mRNA vaccine formulations over time. The ongoing research and development must continue to achieve breakthroughs in this emerging direction. In addition to developing formulations for combination vaccines, it is highly desirable to optimize their delivery and ensure compliance with regulatory standards to maintain safety in their administration.

### 3.2. Recombinant Combination Vaccines

These are new immunization tools that combine the benefits of combination vaccines with recombinant DNA technology. For example, COVID-19 and influenza combination vaccines demonstrated good tolerance and immunogenicity, with dose adjustments resolving antigen interference and achieving responses like individual vaccines [57]. Two investigational combination vaccines have been granted a fast-track designation. Fluzone High-Dose/Novavax combining Fluzone High-Dose, a trivalent inactivated influenza vaccine (H1N1, H3N2, B/Victoria D) and the Novavax COVID-19 vaccine, a recombinant spike protein of the original (Wuhan Hu-1) SARS-CoV-2 with Matrix-M adjuvant. Flublok/Novavax combines Flublok, a quadrivalent recombinant influenza vaccine (H1N1, H3N2, B/Victoria B/Yamagata), with the Novavax COVID-19 vaccine [58]. An analysis of various studies revealed that COVID-19 administration might increase when combined with influenza, leveraging the accustomed routine of annual shots of influenza [59]. Preclinical studies of various candidates have shown that SARS CoV-2 and influenza combinations elicit strong immune responses without compromising the efficacy of either component. The flu–COVID-19 combo combining influenza HA proteins with the SARS-CoV-2 S protein, formulated with AddaVax, has shown encouraging results in K18-hACE-2 transgenic mice. It has shown protective immune responses comparable with individual flu or COVID-19 vaccines [60]. In addition to flu and COVID-19, recombinant vaccines are also being explored for other diseases. A recombinant vaccine combining diphtheria toxin CRM197 and tetanus toxin fragment C (RTCV) induced high levels of IgG antibodies, similar to those observed with traditional DTaP vaccines, indicating its potential as an adult booster [61]. While combination recombinant vaccines offer a promising strategy for enhancing public health, their development faces several challenges. Balancing efficacy, safety, and stability is critical, particularly for vaccines targeting multiple pathogens. The careful selection of adjuvants and antigens has been shown to improve the efficacy of recombinant vaccines against a wide range of infectious diseases [62,63,64].

### 3.3. Live Attenuated Combination Vaccines

Development of attenuated combination vaccines rely on applying weakened pathogens for achieving an immune response. It can be potentially beneficial for raising specific as well as broader immunity against multiple infections. For instance, the dCoV live attenuated COVID-19 vaccine was developed to protect children. It induced neutralizing antibodies and provided protection against Wuhan-like and delta variants. It maintained protection when integrated with the measles–rubella vaccine (MR–dCoV), with no interferences with MR-induced immunity. These findings support its potential for early immunization among children without additional injections [65]. The MMR vaccine, known for preventing measles, mumps, and rubella, is potentially effective against COVID-19 by inducing trained innate immunity. It has been reported that live attenuated vaccines stimulate the production of long-lived myeloid-derived suppressor cells, which play a role in inhibiting septic inflammation and preventing fatality. Like BCG, MMR is being explored for its potential role in enhancing broad immune defense against COVID-19 [66]. Studies revealed that varicella vaccine coverage heightened after its combination with MMR. Most children received MMRV (96%), with only a few opting for separate vaccines. This demonstrates that parents prefer combination vaccines, resulting in improved coverage [67]. Another study reported that the bivalent recombinant LAIV expressing pneumococcal surface Antigen A (PspA) induced IgG production against both pathogens. It provided full protection against influenza and almost partial protection against bacterial coinfection in mice, demonstrating the potential for combined protection against viral and bacterial pathogens [68]. Animal studies of the bivalent live attenuated Metavac^®^-RSV vaccine have revealed promising immune responses against respiratory syncytial virus and human metapneumovirus [69]. These developments highlight the significant potential of live attenuated combination vaccines to combat emerging infections. However, it is essential to consider the non-specific immune responses of live attenuated combination vaccines for optimizing their formulation and maximizing effectiveness.

### 3.4. Virus-Based Combination Vaccines

Viral vectors provide an innovative base for the development of combination vaccines. Such vaccines have demonstrated the production of effective immune responses while maintaining safety [70,71]. An important example using this approach is the development of a vaccine from a chimpanzee adenovirus Serotype 68 vector (AdC68), encoding the RBD of coronavirus spike protein combined with the hemagglutinin stalk of the influenza virus [72]. A vesicular stomatitis virus (VSV) vector containing SARS-CoV-2 spike protein or RBD and influenza virus’s conserved M2 has been developed [73]. It has shown effective humoral and cell-mediated immunity against these viruses. These examples highlight the significance of AdC68-based and VSV-based bivalent vaccines to combat respiratory infections. Another viral vector vaccine composed of measles virus vectors fused with HPV antigens has shown protection against both viruses [74]. Although significant development has been achieved, pre-existing immunity may negatively influence the efficacy of viral vector-based vaccines, which needs to be addressed for fully exploiting their potential [75]. Influenza virus-based vector vaccines carrying SARS-CoV-2 antigens are emerging as promising candidates. For instance, an LAIV vector expressing SARS-CoV-2 T-cell epitopes *NA* or *NS1* genes were generated. While intranasal immunization in mice did not induce significant T-cell responses, likely due to influenza epitopes’ immunodominance. It protected hamsters from both influenza and SARS-CoV-2 variants. These findings support the LAIV vector as a potential platform for a bivalent influenza–SARS-CoV-2 vaccine [76]. In another similar study, an NS1-deleted influenza virus vector expressing the RBD of SARS-CoV-2 demonstrated protection against SARS-CoV-2 variants in animal studies. It induced innate immunity, trained immunity, and localized memory T-cells, suggesting its potential as broad-spectrum vaccine [77]. Recently, a chimeric vaccine targeting influenza and SARS-CoV-2 has been reported. It has been produced by inserting SARS CoV-2 S and N segments into the influenza genome, specifically in an NA of a cold-adapted H3N2 strain of influenza. The vaccine was administered to non-human primates intranasally. It was found to be safe and immunogenic in non-human primates, supporting its potential for clinical trials [78].

Beyond viral vectors, virus-like particles (VLPs) provide an interesting platform to develop combination vaccines. For instance, combining influenza VLPs with SARS-CoV-2 spike RBD and GM-CSF as adjuvant has shown strong antibody responses and provided protection against targeted viruses in mouse studies. Vaccinated mice showed lower virus titers in the lungs. These investigations highlight the potential of hybrid VLPs as a promising candidate to prevent respiratory infections [79]. VLP-based strategies have also been explored for other viruses. VLPs containing epitopes of varicella-zoster virus and Enterovirus 71 have demonstrated the capability to neutralize both viruses and provide protection against infection [80]. A VLP-based combination vaccine, AP205, presenting antigens from dengue and Zika viruses, has generated strong humoral immune responses and neutralized these viruses. Such findings have highlighted the potential of VLP platforms for developing combination vaccines for diverse infections [81].

## 4. Advantages of Combination Vaccines

**Figure 2:**
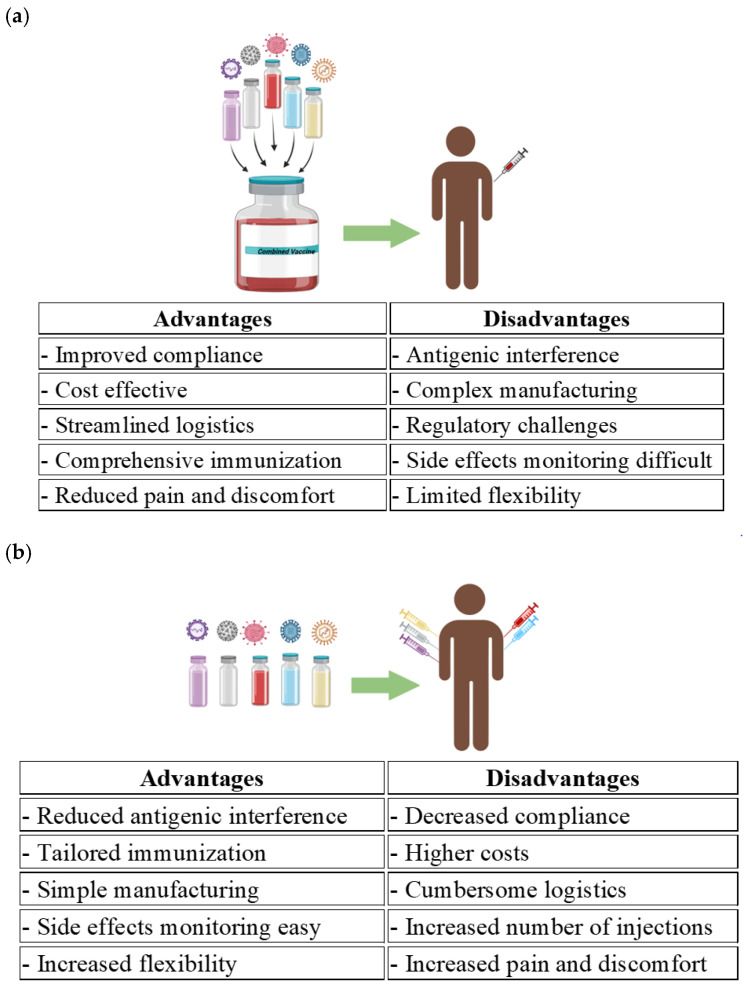
Comparative analysis of combination versus individual vaccines. (**a**) Advantages and disadvantages of combination vaccines; (**b**) advantages and disadvantages of individual vaccines. Created with BioRender, available from https://www.biorender.com/ (Accessed on 20 January 2025).

Combination vaccines, as a cutting-edge and emerging technology, have a lot to offer for effective immunization. Better immunogenicity and extended protection against diverse infections are notable features associated with combination vaccines. These are particularly beneficial in reducing the burden of vaccination in a more simplified way. The advantages of combination vaccines in comparison with individual vaccines are highlighted in Figure 2. Combining influenza and SARS-CoV-2 can improve vaccine compliance and reduce respiratory infections. It will reduce severe outcomes like hospitalization and patient influxes during the respiratory illness season. It minimizes the number of immunization visits and injections [82]. These advantages encourage widespread vaccination to more of the population while reducing the burden at health care facilities, which is particularly beneficial for resource-limited regions. The combination of vaccines decreases pain through fewer injections. For example, a combination of hexavalent DTaP–IPV–Hib–HepB is administered in one shot, while its administration separately needs six injections. According to CDC, fewer injections lead to higher compliance. Combination vaccines are particularly beneficial for those with needle phobia [83].

Key benefits of combination vaccines include their capability of generating broader immunity through incorporation of multiple antigens. It mimics the natural encounter of our immune system with multiple antigens. The immune system is capable of processing multiple antigens simultaneously. Even with combination vaccines, only a tiny fraction of the immune capacity is utilized. Simultaneous administration of 11 vaccines would engage 0.1% of the immune system. This indicates that combination vaccines do not overload the immune system [84,85]. The combination vaccines activate the immune system by presenting various antigens, activating B- and T-cells that are crucial for effective vaccine development. For instance, the trivalent vaccine comprising NoV GII-4 and GI-3 VLPs and RV rVP6 produced cross-reactive antibodies and T-cell immune responses against both viruses. The results encourage further advancement of the NoV and RV combination vaccines for human use [86]. Other multivalent vaccines developed to fight against multiple viral infections caused by the Ebola, Sudan, Marburg, and Lassa viruses has elicited immunity in preclinical studies [70]. This highlights the strength of combination vaccines to provide protection against multiple viruses. In another interesting development, the MV130 poly-bacterial mucosal vaccine has been found to be effective against a range of respiratory infections [87]. Advancements in the field of reverse vaccinology and immunoinformatics have been found very useful to quickly configure the composition of combination vaccines to address the challenges of emerging pathogens and variants [88,89]. This concept can be particularly useful to design multi-epitope vaccines composed of multiple antigens eliciting diverse immune responses. A combination vaccine for targeting coinfection with SARS-CoV-2 and monkeypox has demonstrated great potential against emerging threats [89]. Through the prevention of multiple infections simultaneously, combination vaccines reduce the reliance on antibiotics. This is greatly needed for addressing the growing challenge of antibiotic resistance, as it helps reduce the spread of antibiotic-resistant bacteria [3].

## 5. Challenges and Future Prospects

Aside from the unique advantages and great potential of combination vaccines, certain bottlenecks hinder their widespread applications. Combination vaccines need the separate production of multiple antigens, each produced according to its specific needs and quality control standards. These antigens are combined into a single formulation, making the overall production process more complex and cumbersome than a single vaccine. Another concern is the interdependency of antigens. Suppose that an antigen is experiencing production issues and is not available on time. In that case, the entire production process may be halted, leading to a shortage in the supply chain to meet global needs. For instance, the globally used DTaP vaccine, which serves as the backbone for the development of the hexavalent vaccine, involves several parallel tasks to produce distinct antigens and the adjuvant before formulating the final vaccine product [90]. The combination vaccines receive approval after confirmation that the combination has not reduced efficacy and is not associated with an increase in reactogenicity compared with the individual vaccines [37]. Quality control takes almost 70% time of the entire manufacturing cycle [90]. The complexity of production limits the number of manufacturers capable of large-scale production, increasing the risk of global supply shortages. Addressing these obstacles is vital. Achieving this goal requires technological breakthroughs that may be beyond the reach of low- and middle-income nations [91,92]. Moreover, regulatory policies and commercial barriers significantly impact the development and implementation of combination vaccines. The limitation of high production costs and low returns potentially discourages investment and innovation in this field [15].

Antigenic interference is another key challenge associated with combination vaccines. It may occur when an antigen’s immune response influences another antigen’s response. It could potentially reduce the efficacy and safety of combination vaccines as compared with individual vaccines. For instance, a combination vaccine addressing the Hantaan and Puumala viruses simultaneously has demonstrated reduced efficacy for infections caused by the Hantaan virus [93]. Similarly, the development of a combination vaccine for enterotoxigenic *Escherichia coli* and Shigella has been challenging in terms of eliciting robust immune responses against each infection without interference [94]. Recently reported clinical trials of COVID-19 and influenza combination vaccines demonstrated immunogenicity and antibody responses comparable with the individual rS vaccine (NVX-CoV2373) and licensed influenza vaccines. Evidence of modest interference between the rS of SARS-CoV-2 and HA components was also reported, which was overcome by adjusting the antigen doses. A higher rS dose (>20 µg) overcame HA interference in a dose-dependent manner, while a lower, intermediate HA dose overcame rS interference, achieving immune responses comparable with standalone vaccines [57]. Novavax utilized AI-driven Design of Experiments (DoE) modeling to select optimal doses for its CIC vaccine. This strategy reduced the antigen content by up to 50% while maintaining robust immunogenicity. Minimizing antigen interference makes the vaccine responses comparable with those of standalone vaccines. It reflects a broader industry trend toward using AI in vaccine development [95]. The compatibility issue may arise from the key components or additives such as preservatives or adjuvants. The most likely are from physiochemical factors during formulation that may affect stability and potency, biological interference among attenuated agents, or immunological interference, as seen preclinically or clinically [96]. Future advancements may enable more vaccine combinations by leveraging mRNA, viral vector, and recombinant platforms. The flexibility of mRNA vaccines allows the incorporation of multiple antigens, simultaneously targeting a wide range of pathogens. Innovative viral vectors optimized for robust immunogenicity and capable of addressing pre-existing immunity are promising. Recombinant platforms reduce the risk of antigen interference through precise antigen expression. Additionally, researchers are exploring the capacity of various platforms for maximum antigen inclusions without compromising efficacy and safety.

The immunization schedule of vaccines needs to be carefully planned to guarantee an adequate immune response. Ensuring that the immune system is effectively stimulated by each antigen, maximizing vaccine efficacy, and avoiding potential interference are essential. Timely administration and adherence to immunization schedules could be particularly challenging in a population at high risk of missed appointments [97]. Lack of awareness, socioeconomic barriers, logistic issues, and limited access to health care facilities may result in missed or delayed vaccination. The acceptance rate of combination vaccines may be increased by addressing the misconception that the administration of combination vaccines could overburden the immune system. Health care workers should be trained to observe and manage side effects associated with combination vaccines. This will enhance combination vaccines’ safety and achieve public confidence. In order to ensure that the benefits outweigh the risks, combination vaccines require a thorough analysis of any adverse events [97]. Maintaining immunization records is important to ensure the effectiveness of health care systems, as it ensures timely vaccinations and enables disease surveillance and outbreak control. There is a need for improvements in systems to ensure the transfer of accurate and complete information on combination vaccines into the medical records and immunization registries to avoid ambiguities in the vaccines’ naming. The high production cost of combination vaccines can be a barrier locally, specifically for regions like Africa, where vaccine production infrastructure is still developing [92]. Coordinated efforts, including investments in technology, state of the art facilities, and uniform regulatory frameworks, can lead to sustainable and economical production of combination vaccines. Policy updates underscore the importance of a unified approach involving regional consultations to identify priorities, modernizing regulatory frameworks to reflect the advantages of combination vaccines, and increasing financial commitments while strengthening the support of global health organizations such as the WHO and GAVI. Overcoming these challenges is imperative to secure the success of existing combination vaccines and drive their innovation in the coming years.

## 6. Conclusions

Combination vaccines are the cornerstone of innovations offering a single, convenient solution to address multiple pathogens. Modernization in mRNA vaccines has opened new possibilities in the field of combination vaccines. This emerging trend has been very useful in terms of reducing injection schedules, saving time and resources for both patients and health care providers. Addressing multiple infections with one formulation is particularly beneficial for resource-constrained areas and those with high disease burdens. Aside from the benefits, developing combination vaccines for a wide range of infections is currently challenging. Combining antigen demands addressing issues of compatibility, stability, and compliance with stringent regulatory requirements. Therefore, further research is required to address such constraints and harness the full potential of combination vaccines in combating various infectious threats.

## Figures and Tables

**Table 1:** Combination vaccines for immunization [43,44].

Combination	Brand Name	Vaccine	Coverage
Hexavalent	Infanrix hexa^®^	DTaP–HepB–Hib/IPV	Diphtheria, tetanus, acellular pertussis, poliovirus, Haemophilus influenzae Type b, and Hepatitis B
	Vaxelis^®^	DTaP–IPV–Hib–HepB	
	HEXASIIL^®^	DTwP–HepB–IPV–Hib vaccine	Diphtheria, tetanus, whole-cell pertussis, Hepatitis B, poliovirus, Haemophilus Influenzae Type B
Pentavalent	Pediarix™	DTaP–HepB–IPV	Diphtheria, tetanus, acellular pertussis, Hepatitis B, poliovirus
	Pentacel™	DTaP–IPV–Hib	Diphtheria, tetanus, acellular pertussis, poliovirus, Haemophilus influenzae Type B
Tetravalent	Kinrix™	DTaP–IPV	Diphtheria, tetanus, acellular pertussis, poliovirus
	TriHIBit	DTaP–Hib	Diphtheria, tetanus, acellular pertussis, Haemophilus influenzae Type B
	ProQuad^®^	MMRV	Measles, mumps, rubella, varicella
Trivalent	Tripedia™ Daptacel™ Infanrix™	DTaP	Diphtheria, tetanus, acellular pertussis
	Adacel™	DTaP (Adult)	
	Boostrix™		
	M-M-R^®^ II	MMR	Measles, mumps, rubella
	PRIORIX^®^		
Bivalent	COMVAX^®^	Hib–Hep B	Haemophilus influenzae Type B, Hepatitis B
	Twinrix^®^	HepA–HepB (Adult)	Hepatitis A, Hepatitis B

**Table 2:** Overview of the emerging candidates for combination vaccines.

Platform	Target	Antigens	Trial Phase	CTN	Sponsor (Vaccine)
mRNA	Influenza and SARS-CoV-2	HA of Influenza A (H1N1, H3N2), and Influenza B (Victoria and Yamagata lineage), RBD and NTD of spike protein of SARS-CoV-2 omicron BA.4/BA.5 subvariants	**1, 2, 3**	NCT05827926 NCT06097273 NCT06694389 NCT06508320	Moderna (mRNA-1083)
mRNA	Influenza and SARS-CoV-2	HA of Influenza A (H1N1, H3N2), and Influenza B (Victoria and Yamagata lineage), S-2P prefusion stabilized spike protein of SARS-CoV-2 original Wuhan-Hu-1	**1, 2**	NCT05375838	Moderna (mRNA-1073)
mRNA	Influenza and SARS-CoV-2	HA of Influenza A (H1N1, H3N2), and Influenza B (Victoria and Yamagata lineage), spike protein of SARS-CoV-2 original Wuhan-Hu-1 and omicron BA.4/BA.5 subvariants	**1, 2**	NCT06696734	BioNTech SE (qIRV(22/23)/ bivalentBNT162b2)
mRNA	Influenza and SARS-CoV-2	HA of tIRV (H1N1, H3N2, Victoria lineage) or HA of qIRV (H1N1, H3N2, Victoria and Yamagata lineage), spike protein of SARS-CoV-2 original Wuhan-Hu-1 and Omicron BA.4/BA.5 subvariants	**3**	NCT06178991	BioNTech SE (Combination A, Combination B)
mRNA	Influenza, RSV, and SARS-CoV-2	mRNA-1045: HA of 4 Influenza A (H1N1, H3N2), and Influenza B (Victoria and Yamagata linages), prefusion fusion protein of RSV; mRNA-1230: an additional spike of SARS-CoV-2	**1**	NCT05585632	Moderna (mRNA-1045, mRNA-1230)
mRNA	hMPV and PIV3	Fusion protein of hMPV, fusion protein of PIV3	**1**	NCT04144348 NCT03392389	Moderna (mRNA-1653)
Viral vector	Ebola and Marburg	Chimpanzee adenoviral vector containing GP of Sudan ebola, chimpanzee adenoviral vector containing Marburg Angola GP	**1**	NCT04723602	Albert B. Sabin vaccine Institute (cAd3-EBO-S and cAd3 Marburg)
Nanoparticle vaccine	Influenza and SARS-CoV-2	SARS-CoV-2 recombinant spike nanoparticle, quadrivalent HA nanoparticle influenza combination vaccine with Matrix-M adjuvant	**1, 2**	NCT04961541 NCT05519839	Novavax (qNIV/CoV2373)
Recombinant	Influenza and SARS-CoV-2	Recombinant influenza vaccine + adjuvanted recombinant COVID-19 vaccine	**1, 2**	NCT06695130	RIV + rC19
Inactivated/recombinant	Influenza and SARS-CoV-2	Inactivated influenza vaccine + adjuvanted recombinant COVID-19 vaccine	**1, 2**	NCT06695117	IIV-HD + rC19

Abbreviations: HA, hemagglutinin; NTD, N-terminal domain of SARS-CoV-2 spike protein; RBD, receptor binding domain of SARS-CoV-2 spike protein; RSV, respiratory syncytial virus; hMPV, human metapneumovirus; PIV3, Parainfluenza virus Type 3; GP, glycoprotein; tIRV, trivalent mRNA-based influenza vaccine; qIRV, quadrivalent mRNA-based influenza vaccine; RIV, recombinant influenza vaccine; IIV-HD, high-dose inactivated influenza vaccine.
